# Fabrication and Validation of a 3D Portable PEGDA Microfluidic Chip for Visual Colorimetric Detection of Captured Breast Cancer Cells

**DOI:** 10.3390/polym15153183

**Published:** 2023-07-27

**Authors:** Mingyi Guo, Yan Deng, Junqiu Huang, Yanping Huang, Jing Deng, Huachang Wu

**Affiliations:** 1 College of Food Science and Technology, Sichuan Tourism University, Chengdu 610100, China; guomy@sctu.edu.cn (M.G.);; 2 College of Bioengineering, Chongqing University, Chongqing 400044, China; 3 College of Bioengineering, Sichuan University of Science and Engineering, Zigong 644005, China

**Keywords:** poly(ethylene glycol) diacrylate, hydrogel microfluidic chip, colorimetric sensor array, breast cancer, detection, cancer

## Abstract

To guide therapeutic strategies and to monitor the state changes in the disease, a low-cost, portable, and easily fabricated microfluidic-chip-integrated three-dimensional (3D) microchamber was designed for capturing and analyzing breast cancer cells. Optimally, a colorimetric sensor array was integrated into a microfluidic chip to discriminate the metabolites of the cells. The ultraviolet polymerization characteristic of poly(ethylene glycol) diacrylate (PEGDA) hydrogel was utilized to rapidly fabricate a three-layer hydrogel microfluidic chip with the designed structure under noninvasive 365 nm laser irradiation. 2-Hydroxyethyl methacrylate (HEMA) was added to the prepolymer in order to increase the adhesive capacity of the microchip’s surface for capturing cells. 1-Vinyl-2-pyrrolidone (NVP) was designed to improve the toughness and reduce the swelling capacity of the hydrogel composite. A non-toxic 3D hydrogel microarray chip (60 mm × 20 mm × 3 mm) with low immunogenicity and high hydrophilicity was created to simulate the real physiological microenvironment of breast tissue. The crisscross channels were designed to ensure homogeneous seeding density. This hydrogel material displayed excellent biocompatibility and tunable physical properties compared with traditional microfluidic chip materials and can be directly processed to obtain the most desirable microstructure. The feasibility of using a PEGDA hydrogel microfluidic chip for the real-time online detection of breast cancer cells’ metabolism was confirmed using a specifically designed colorimetric sensor array with 16 kinds of porphyrin, porphyrin derivatives, and indicator dyes. The results of the principal component analysis (PCA), the hierarchical cluster analysis (HCA), and the linear discriminant analysis (LDA) suggest that the metabolic liquids of different breast cells can be easily distinguished with the developed PEGDA hydrogel microfluidic chip. The PEGDA hydrogel microfluidic chip has potential practicable applicability in distinguishing normal and cancerous breast cells.

## 1. Introduction

Breast cancer is one of the most severe diseases that pose a threat to human health at present. According to the latest estimates on the global burden of cancer from the International Agency for Research on Cancer (IARC), the breast cancer burden is estimated to have risen by 2.26 million new cases and 0.685 million deaths in 2020 [1]. It accounts for more than 11.7% of all newly diagnosed cancer cases and has overtaken lung cancer as the most common cancer type [2]. Typical detection methods for breast cancer rely on large-scale instruments and equipment, with high requirements relating to professional and technical operation, as well as high costs [3]. Therefore, there is an urgent need to develop effective, inexpensive, and universal detection methods. At the same time, figuring out how to achieve simple, rapid, accurate, and low-cost detection has remained the main focus of breast cancer diagnosis research [4].

To address the above issue, microfluidics may be employed, which is a set of technologies and sciences used for manipulating nanoliter volumes of fluids in channels with dimensions measured in tenths or even hundredths of micrometers [5,6]. Chip technology based on microfluidic science has great advantages in terms of its low reagent consumption, low requirement of sample preparation, and excellent cell bioactivity. In particular, the chips can be used to closely simulate the physiological microenvironment and cell interactions in vivo [7,8]. 2-Hydroxyethyl methacrylate (HEMA) and polyethylene glycol (PEG), as bases for hydrogel chips, are broadly studied materials [9,10] and represent novel tools for biomedical and tissue engineering applications due to their non-toxic and non-immunogenic characteristics, excellent biocompatibility, and tunable physical properties as well as their high surface modification capacity [11,12]. PEG-based hydrogel scaffolds are capable of supporting three-dimensional (3D) cell and tissue growth within crosslinks [13]. By substituting terminal hydroxyl groups with acrylates to form poly(ethylene glycol) diacrylate (PEGDA), the polymers can be crosslinked into a three-dimensional polymer network with high permeability and water content [14,15]. This is a more suitable scaffold for use in cancer diagnosis, tissue engineering, and regenerative medicine [16,17,18]. A 3D hydrogel microfluidic chip has a microarchitecture that was specifically designed to reproduce the in vivo environment, and it can thus yield a physiologically representative response resulting in the improved functionality of the tissue [19,20,21]. In the presence of a photo-initiator and UV light, a PEGDA hydrogel can be rapidly formed at room temperature with a low energy input [22]. Furthermore, the produced chip can be manipulated into complex shapes and made to have different mechanical strengths by controlling the time and distance of the photopolymerization [23]. PEGDA can also be customized to incorporate a variety of biological molecules with particular functions into the predetermined three-dimensional structure depending on the purpose [24,25]. PEGDA’s crosslinked structure provides substrates with spatially patterned, tunable mechanical properties along different gradients [26,27,28]. A PEGDA hydrogel can be used to build a research platform for use in microfluidic cell biology in order to realize real-time observations and detection [19,29].

A PEGDA hydrogel microfluidic chip has the properties of simple construction, fast formation, high precision, a low cost of the cell culture, and the real-time detection of metabolites [30,31]. Cells can be captured and cultured in the hydrogel microfluidic chip and show normal activity. HEMA has been added to the PEGDA prepolymer in order to increase the adhesive capacity of the microchip’s surface for cells [32,33]. 1-Vinyl-2-pyrrolidone (NVP) was designed to improve the toughness and yield strength and to reduce the swelling capacity of hydrogel composites [34]. NVP was also utilized as a dissolvent in the photo-initiator process. This study provides a method for effectively distinguishing different types of breast cancer cells from normal breast cells by detecting their metabolism characteristics. The hydrogel microfluidic device provides a high-efficiency and high-sensitivity method for capturing breast cancer cells in specific areas, permitting purposeful stimulation and observation. In the present study, the ultraviolet polymerization characteristic of the PEGDA hydrogel material was exploited to fabricate a desirable microfluidic chip via the photopolymerization method. This low-cost, portable, and easily fabricated PEGDA hydrogel microfluidic chip has potential applications in breast cell culture, analysis, and medical detection.

## 2. Materials and Methods

### 2.1. Chemicals

PEGDA (Mn = 575) containing 400~600 ppm MEHQ as an inhibitor, HEMA, and NVP stabilized with N,N’-di-secbutyl-1,4-phenylenediamine were used as crosslinking agents. NVP stabilized with N,N’-di-secbutyl-1,4-phenylenediamine, was used as the crosslinking agent. All reactants were purchased from Sigma–Aldrich Co. (Saint Louis, MO, USA). Irgacure 2959 (2–hydroxy-4′-(2-hydroxyethoxy)-2-methylpropiophenone) was used as the photo-initiator agent. The process was as follows: Firstly, the PEGDA prepolymer solution was prepared. The mixture of the prepolymers PEGDA, HEMA, and NVP was prepared with a volume ratio of 6:4:0.5. Then, the photo-initiator Irgacure 2959 (AR, 0.3% *w*/*v*) was added to the solution followed by intensive mixing. The obtained mixture was vacuumed to remove air bubbles. Free radicals of the photo-initiator were generated to promote the polymerization of the prepolymer monomers under UV light irradiation. The desired PEGDA network was fabricated in 2 h. 

### 2.2. The Microfluidic Chip Design

**Figure 1:**
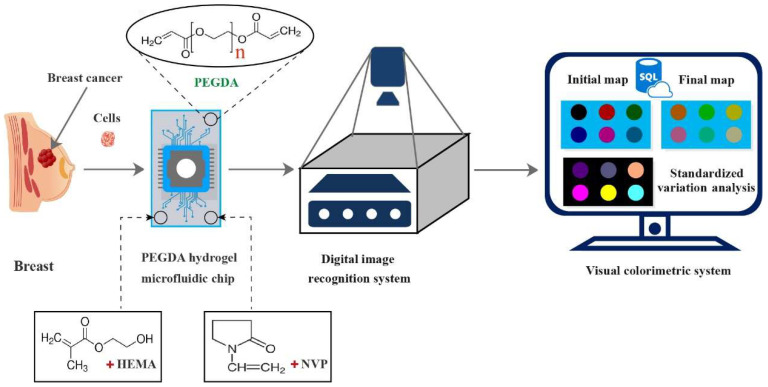
The designed test flow of the microfluidic chip used for detecting the metabolic substances of breast cells.

**Figure 2:**
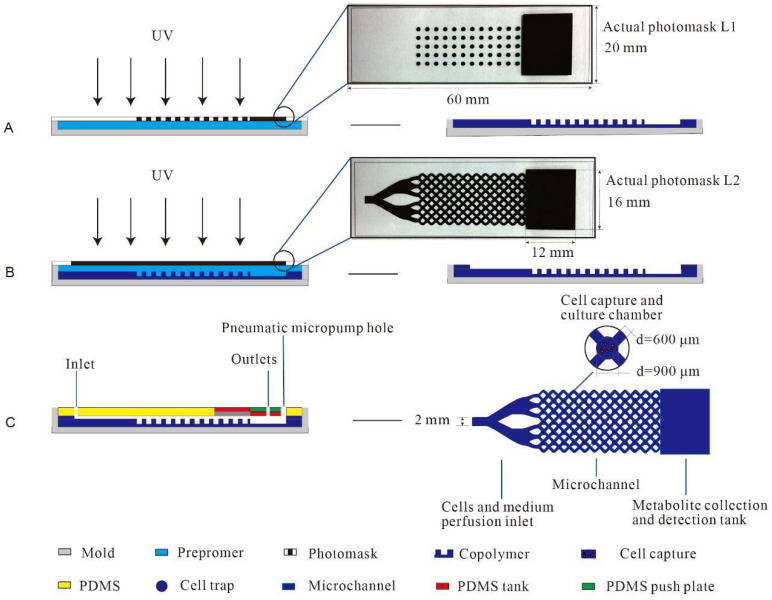
Illustration of the fabrication of the PEGDA hydrogel microfluidic chip via photopolymerization to obtain the first layer of the microwell structure (**A**); the second layer of the channel (**B**) and the detailed structure (**B**). The microchannel structure was clearly distributed within the chip (**C**).

The test flow of the microfluidic chip used for assessing the metabolic substances of breast cells is shown in Figure 1. The most central and important process node is the design and fabrication of the PEGDA hydrogel microfluidic chip. Firstly, CorelDRAW graphics software was adopted to design the microfluidic chip mask. The chip mask consisted of three parts, as shown in Figure 2. The first part was composed of a circular porous microarray (microwell; D = 900 μm; 5 × 12) and a 12 mm × 16 mm rectangular structure for metabolite gathering and detection. The main function of the microwell was to separate and capture the cells evenly, which can effectively prevent fluid shear stress injury in the cells and help reflect their true shapes. The second part comprised a precise network with a fluid channel structure, in which an elaborate staggered channel (D = 600 μm) was designed to homogeneously distribute the cells with appropriate densities. The second part also had the same 12 mm × 16 mm rectangular structure. The medium was evenly distributed via injection to ensure its effective capture in the crisscross microchannels. The third part had a grooved structure and was equipped with a pneumatic microhole in order to improve the flow rate of the medium and metabolites. An airtight cover made of poly(dimethylsiloxane) (PDMS) was used [35]. The three units described above together comprised the airproof microfluidic container.

### 2.3. The Microfluidic Chip Fabrication

**Figure 3:**
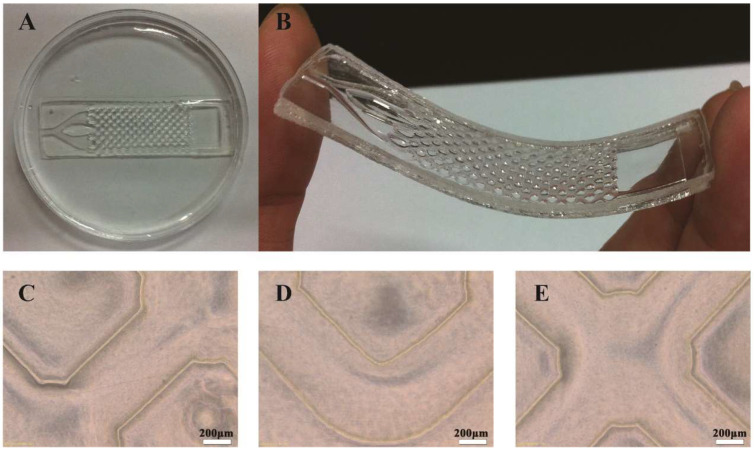
The final product of the PEGDA hydrogel microfluidic chip (**A**,**B**), and the microstructure of the chip under a microscope (**C**–**E**).

The PEGDA hydrogel microfluidic chip was fabricated using the following process, as shown in Figure 2. With the use of a glass mold as the substrate, the first layer of the mask and the glass slide were closed to form an airtight cavity with specific dimensions (height: 1 mm). Next, the prepolymer solution was injected into the cavity slowly using a syringe. The whole mold was then exposed to long-wavelength UV light (10 mmW/cm^2^, 365 nm) for 30 min, and the entire reaction process took place under nitrogen protection. After the first layer mask was gently removed, the first layer of the microwell structure was obtained. By increasing the height of the mold, the second layer of the microchannel structure could also be created with similar methods. The hydrogel microfluidic chip was placed into PBS buffer, 75% ethanol, and tri-distilled water to remove the unreacted prepolymer and was then dried naturally. The airtight part of the PDMS cover was subsequently placed on top to cover the hydrogel. Then, the desired chip was completed. Photographs of the PEGDA hydrogel microfluidic chip and the microwell structure are clearly shown in Figure 3A,B.

### 2.4. Cell Culture

**Figure 4:**
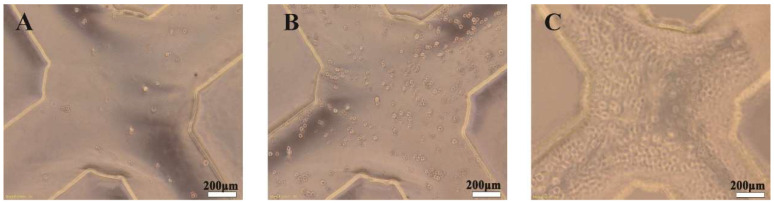
Cells were automatically captured in the microwell structure and uniformly distributed. Cell seeding (**A**), cell proliferation (**B**), and after cell culturing (**C**).

Prior to the inoculation of the breast cancer cells, the PEGDA hydrogel microfluidic chip was sterilized with ultraviolet irradiation for 3 h and with a 75% ethanol solution for 2 h. After soaking in the sterilized PBS for 1 h, it was inoculated and transferred to a thermostatic incubator (Thermo Fisher Scientific Inc., Waltham, MA, USA) and kept in a standardized and humidified atmosphere for 72 h for incubation at 37 °C and in 5% CO_2_. The basic culture conditions were 1640 medium (Gibco, Life Technologies Corporation, Carlsbad, CA, USA) supplemented with 10% fetal bovine serum (Gibco, Life Technologies Corporation, Carlsbad, CA, USA), 100 U mL^−1^ penicillin, and 100 U mL^−1^ streptomycin. The cell suspension was firstly inoculated at the entrance of the chip using a pipette with a density of 1 × 10^6^/mL. The cells were then captured spontaneously in the microwells. The microfluidic chip was placed in a culture dish and then in the incubator for 4 h of adhesion before non-adherent cells were rinsed off. Then, the metabolic fluid in the rectangular groove (collecting tank) was collected. Figure 3C–E shows the microscope observations of the chip’s microwell structure before cell seeding. And Figure 4A–C shows the microscope observations of the chip’s actual situation after cell culturing. MCF-7, human breast adenocarcinoma cells, were selected as the experimental sample to display the capture effect.

### 2.5. The Detection of Metabolite Characteristics

Sixteen chemical pigments and porphyrins, including water-soluble porphyrin substituted with sulfonic groups and metal porphyrin derivatives, were selected as the indicators to detect the metabolites of the cells based on previous studies by this group [36,37,38]. The sensitive dots (4 × 4 sensor assay) were fabricated by placing 1 μL of each indicator on the polyvinylidene fluoride (PVDF) membrane. The indicators of the biosensor array were prepared using sol–gel formulations, which are essentially impervious to changes in relative humidity. The fabricated PVDF membrane was placed into the metabolite collection tank to detect the cell metabolism characteristics. Human breast cancer cells MCF-7, HCC38, ZR-75-30, BT-20, and MDA-MB-468 and human normal breast cells MCF-10A, MDA-kb2, Hs578Bst, HMLE, and HTB-125 were purchased from the Chinese Academy of Science’s cell bank for this experiment. Images of the sensor array were captured before and after (10 min later) detection. By comparing the two images of the array, RGB (red, green, and blue) color change values of −255 to 255 were obtained for statistical analysis.

### 2.6. Statistical Analysis

Statistical analyses were conducted using R (v 4.3.1) (https://www.r-project.org/ (accessed on 16 June 2023)) with custom scripts with the available packages in this project. Principal component analysis (PCA) was used to examine the relationship between the composition and the variability of cell metabolites. PCA is an unsupervised technique that reduces the dimensionality of the original data matrix, retaining the maximum amount of variance. Linear discriminant analysis (LDA) is a supervised technique used for classification purposes. Hierarchical cluster analysis (HCA) was also used to analyze the RGB database of the samples. This clustering technique comprises an unsupervised chemometric procedure that involves the measurement of either the distance or similarity between objects to be clustered. Objects are grouped in clusters in terms of their nearness or similarity. The initial assumption is that the nearness of the objects in the p-space of the variables reflects the similarity of their properties. The statistical significance of the difference between the means of the samples was tested using a two-way analysis of variance (ANOVA) with Duncan’s test (*p* < 0.05) with the IBM SPSS program, version 26.0 (SPSS Inc., Chicago, IL, USA, 2023).

## 3. Results 

### 3.1. Actual Product of the Fabricated PEGDA Hydrogel Microfluidic Chip

As shown in Figure 3C–E, a micrometer-scale microfluidic chip was successfully fabricated. A branching channel structure was established on the chip. In the first layer, 60 circular microwells were arrayed in a 5 × 12 form. The physical size (diameter) of each microwell was 900 μm. The precision of this fabrication process can reach up to dozens of microns with the help of skillful operation. The 5 × 12 microwell array and 12 mm × 16 mm rectangular groove in the first layer exactly align with the second layer’s channel structure and grooves, which aids in subsequent/real-time detection. Figure 3C displays the straight structure of the chip. The curved structure of the chip is shown in Figure 3D. Figure 3E illustrates the cross-micropore structure. These images confirm that there were no deviations in the size of the chip, and it had a neat edge and flat surface. The processing accuracy met the requirements for cell culturing. All structures in the chip were connected, and breast cells can be captured and cultured in its well-organized chamber. Such a chip structure can be used to effectively eliminate fluidic shear stress causing cell injury, as it operates by perfusing the cell medium and flushing with PBS in order to reflect real physiological conditions.

### 3.2. Cell Morphological Observation

It is clear that the cells adhered to the hydrogel substrate (Figure 4) when in their real state and were reproduced in the PEGDA hydrogel microfluidic chip. Acting as an acellular matrix cell culture scaffold in the in vitro environment, the PEGDA chip was shown to be able to successfully mimic the physiological environment of the targeted tissue. The microfluidic chip was also found to be highly effective in protecting the cell from fluidic shear stress.

### 3.3. The Metabolite Characteristics

**Figure 5:**
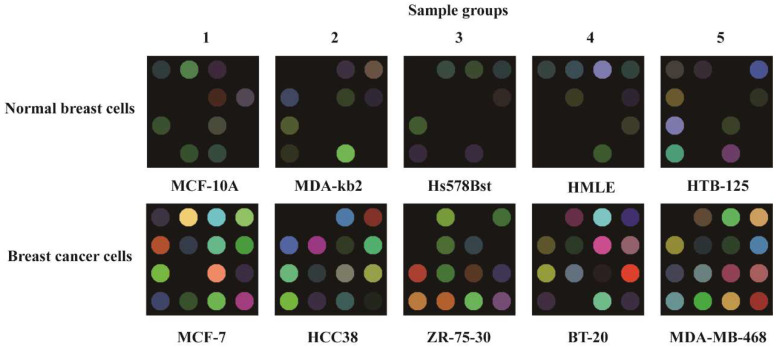
The colorimetric maps of pigment-based arrays for different breast cell metabolites. All results display repetitions, and all experiments were run in quintuplicate.

The two sets of results of the breast cell metabolites showed significant differences, as shown in Figure 5. It is very clear that the colorimetric sensor array registered a unique color change for each breast cancer cell. The visual maps showed distinct differences in the responses of the breast cancer cells (MCF-7, HCC38, ZR-75-30, BT-20, and MDA-MB-468) and the control samples of normal breast cells (MCF-10A, MDA-kb2, Hs578Bst, HMLE, and HTB-125). The breast cancer cells displayed more response points in the colorimetric array maps compared with normal breast cells. 

### 3.4. HCA, PCA, and LDA Analyses

**Figure 6:**
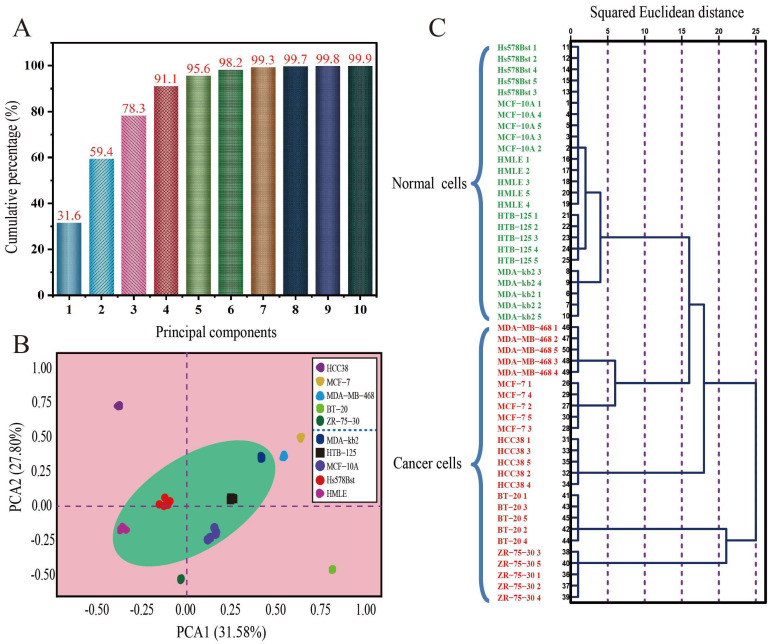
(**A**) Histogram of the cumulative variance contribution values of the first 10 principal components. (**B**) Plot of the first two principal components using PCA with RGB data obtained from the colorimetric sensor. (**C**) HCA dendrogram of the metabolites of 5 different normal breast cells and 5 different breast cancer cells.

The results indicate that the experiments had good repeatability, and the different cells were effectively distinguished. Principal component analysis (PCA) was used to reduce the data dimension, retain low-order principal components, avoid high-order principal components, and maintain the value that had the greatest effect. The top 10 values of the cumulative variance of the largest contribution were selected as values for analysis. The most important 10-dimensional principal components accounted for 99.87% of all the data, as shown in Figure 6A. The PCA of the metabolite data indicates marked clustering of different breast cancer cells in Figure 6B. The first two principal components account for 59.38% of all information. The axes of the two-dimensional PCA diagram account for 31.58% and 27.80% of the plane space. The five parallel samples of the same cell types are not far away from each other. There is also a clear concentration of clusters, and some even overlap. The spatial distance between normal breast cells in the green circle was relatively small, while the spatial distance between the breast cancer cells in the pink outer circle is relatively large, which intuitively shows the difference between the metabolites of the two types of cells. The hierarchical cluster analysis (HCA) results of the metabolic substances of the five groups of breast cancer cells plus the five control normal cells can be seen in Figure 6C, where the clustering effect between the five parallel samples is obvious. The HCA results show that 50 types of metabolic samples were classified within the breast cells based on the squared Euclidean distance (SED). The HCA diagram shows five parallel samples from 10 groups, all of which show obvious aggregation when the SED is less than two, indicating that the differences between the parallel samples were small and the range of change was stable, while both normal breast cells and breast cancer cells underwent group clustering. In particular, when the SED is 4, all of the normal breast cells cluster together, indicating that there was little change in the metabolic fluid of the normal breast cells. When the SED is 25, the two types of metabolic fluid converge, indicating that the differences between the metabolites also increased. Moreover, the HCA results show that clustering the SED of the breast cancer cells revealed a large difference, while the normal breast cells were relatively easily classified. 

## 4. Discussion

### 4.1. Performance Characteristic of the PEGDA Microfluidic Chip

The combination of the PEGDA material’s UV polymerizable function with high-precision masks enabled the efficient production of the hydrogel microfluidic chip. The microscopic observation of the chip indicated consistent dimensions, neat edges, a clear structure, and a smooth surface. Thus, the influence of the instability of a chip’s structure and performance on subsequent culture experiments could be avoided. Consequently, this verified that the processing precision of the PEGDA hydrogel microfluidic chip efficaciously met the demand for cell culture in the detection application.

### 4.2. Effect of Cell Culture on the PEGDA Microfluidic Chip

The results provide evidence that the PEGDA microfluidic chip was able to successfully culture breast cells and effectively protect the cells, mimicking the physiological environment of the targeted tissue. A possible explanation for this might be that the cell suspension fluid flowing through the microchannels on the PEGDA microfluidic chip was automatically directed via gravitational forces into the microholes, filling them with the cell mixture to a depth of 0.3 mm. Subsequently, the cell suspension continued to flow through the microchannels to the next microhole, while any surplus cell suspension spontaneously accumulated in the detection reservoir and was expelled from the chip. Cell adhesion became very challenging due to substantial shear forces in the microchannels. Nevertheless, cells captured in the microholes grew within the designated microarray, facilitating in situ cell observation and analysis, which effectively prevented cell damage caused by fluid shear forces. Therefore, this promoted a uniform cell distribution on the PEGDA chip and facilitated metabolite expression.

### 4.3. Validation of Detection Application of the PEGDA Microfluidic Chip

All breast cells were cultivated via the standardized operating procedure. The 1640 blank medium was used as the control sample. Five groups of parallel experiments were designed, and we obtained data every 8 h, according to the cell growth cycle. All of the experiments using reactive colorimetric sensor arrays for collecting images were run under the same temperature, humidity, and nitrogen control conditions. The results confirmed that the cell metabolites showed significant differences between the normal breast cells and the breast cancer cells in the colorimetric sensor array. Because the breast cancer cells were outside of the normal controlled conditions of the body, their growth became autonomous. The cancer cells transformed and shifted to a state of uninterrupted reproduction and differentiation, thus resulting in metabolic imbalance. Conversely, the growth patterns of the normal breast cells were orderly and uniform. Therefore, the colorimetric array maps of the breast cancer cells showed more response points corresponding to the 16 chemical pigments and porphyrins compared with normal breast cells. PCA is a data analysis method that simplifies datasets by reducing dimensions and retaining low-order principal components while ignoring high-order ones. By preserving the values that contribute significantly to the variance of breast cell samples, it provides a highly reliable measure of data stability. HCA is a multivariate statistical analysis method used to assess the correlation between data and perform classification. It enables the analysis of similarities and differences between experimental samples of breast cells. Smaller squared Euclidean distances indicated smaller differences between the breast cells’ metabolites. Therefore, pattern recognition methods, including PCA and HCA, were used to analyze the RGB database, with changes in red, green, and blue values ranging from −255 to +255. All analyses were assessed against one another and found to contain no errors or misclassifications. The analysis results permitted one to easily discriminate between these different breast cells. The possible reason for this is that the growth and metabolism patterns of breast cancer cells are complex and diverse. The PEGDA hydrogel microfluidic chip supported with a colorimetric sensor array system had the ability to distinguish different cancerous and normal breast cells by capturing metabolites with high sensitivity and accuracy. Compared with traditional frequently used diagnostic approaches such as X-ray, computed tomography (CT), and magnetic resonance imaging (MRI), this study provides a simple, effective, and inexpensive detection method for breast cancer diagnosis.

## 5. Conclusions

In this study, a 3D integrated microfluidic chip was presented for directly trapping and culturing breast cells and detecting metabolites via the photopolymerization of a PEGDA-based hydrogel material. The PEGDA hydrogel microfluidic chip has outstanding advantages in preventing fluidic shear stress injury in cells and helps to reflect their true state. The cell culture experiment demonstrated that the staggered microscale structure can be used to construct uniform cell traps and facilitates subsequent/real-time detection. It also provides a novel method for identifying different breast cancer cells. The method of fabrication is simple, fast, and low-cost. The PEGDA hydrogel microfluidic chip integrated with a colorimetric sensor array system can effectively distinguish different types of breast cells by detecting metabolism characteristics. This work has shown that this 3D PEGDA hydrogel microfluidic chip may represent a label-free, real-time, and cost-effective option for the detection of breast cancer cells with high specificity in cancer research.

## Data Availability

The datasets generated during the current study are available from the corresponding author upon reasonable request.

## Abbreviations

3D	Three-dimensional
HCA	Hierarchical cluster analysis
HEMA	2-hydroxyethyl methacrylate
IARC	International Agency for Research on Cancer
LDA	Linear discriminant analysis
NVP	1-vinyl-2-pyrrolidone
PBS	Phosphate-buffered saline
PCA	Principal component analysis
PDMS	Poly-dimethylsiloxane
PEG	Polyethylene glycol
PEGDA	Poly(ethylene glycol) diacrylate
PVDF	Polyvinylidene fluoride
RGB	Red, green, and blue
SED	Squared Euclidean distance
